# The choice of hypoallergens for fish and peach to develop food allergy specific immunotherapy (the FAST project)

**DOI:** 10.1186/2045-7022-1-S1-O48

**Published:** 2011-08-12

**Authors:** Laurian Zuidmeer-Jongejan, the FAST consortium

**Affiliations:** 1 Academic Medical Center, Experimental Immunology, Amsterdam, Netherlands

## Background

Classical allergen-specific immunotherapy (SIT), using subcutaneous injections with food extracts, may be effective but dangerous due to anaphylactic side-effects. The FAST project (Food Allergy Specific Immunotherapy) aims at the development of safe and effective treatment of food allergies, targeting persistent and severe allergy to fish (cod) and fruit (peach). Both are caused by a single major allergen, parvalbumin (Cyp c 1) and lipid transfer protein (Pru p 3), respectively. FAST will apply hypo-allergenic recombinant major allergens for SIT.

## Methods

Two approaches were evaluated for achieving hypo-allergenicity, i.e. site-directed mutagenesis and chemical modification. Wildtype (wt) natural and recombinant allergens and the hypo-allergens were extensively purified and characterized physico-chemically.. Their stability was tested and allergenicity was compared by CAP-inhibition and histamine release experiments while immunogenicity was tested in T-cell proliferation experiments and rabbit and immunizations.

## Results

For Cyp c 1, the mutant without calcium-binding site showed up to a 1000 times reduced allergenicity, while secondary fold and immunogenicity (human PBMC stimulations/immunization laboratory animals) were retained. Chemically-modified Cyp c 1 demonstrated poorer capacity to stimulate T-cells and immunogenicity in rabbits. For Pru p 3 allergenicity was reduced to a similar extent (~1000-fold) for both variants in which disulfide bridges were disrupted, i.e. either by mutagenesis or by reduction/alkylation. The disruption resulted in loss of alpha-helical secondary structure. Assessment of immunogenicity and stability is still under investigation.

## Conclusion

For Cyp c 1, the calcium-binding mutant will now be produced under GMP regime. For Pru p 3, the final choice for going into this stage still has to be made.

